# Tracking the brain in myotonic dystrophies: A 5-year longitudinal follow-up study

**DOI:** 10.1371/journal.pone.0213381

**Published:** 2019-03-07

**Authors:** Carla Gliem, Martina Minnerop, Sandra Roeske, Hanna Gärtner, Jan-Christoph Schoene-Bake, Sandra Adler, Juri-Alexander Witt, Felix Hoffstaedter, Christiane Schneider-Gold, Regina C. Betz, Christoph Helmstaedter, Marc Tittgemeyer, Katrin Amunts, Thomas Klockgether, Bernd Weber, Cornelia Kornblum

**Affiliations:** 1 Department of Neurology, University Hospital Bonn, Bonn, Germany; 2 Institute of Neuroscience and Medicine (INM-1), Research Center Juelich, Juelich, Germany; 3 Center for Movement Disorders and Neuromodulation, Department of Neurology and Institute of Clinical Neuroscience and Medical Psychology, Medical Faculty, Heinrich-Heine University, Düsseldorf, Germany; 4 German Center for Neurodegenerative Diseases (DZNE), Bonn, Germany; 5 Life and Brain Center, Department of NeuroCognition-Imaging, Bonn, and Department of Epileptology, University Hospital Bonn, Bonn, Germany; 6 Department of Pediatric Kidney, Liver and Metabolic Diseases, Hannover Medical School, Hannover, Germany; 7 Department of Epileptology, University Hospital Bonn, Bonn, Germany; 8 Institute of Clinical Neuroscience and Medical Psychology/ Institute of Systems Neuroscience, Medical Faculty, Heinrich-Heine University, Düsseldorf, Germany; 9 Department of Neurology, Ruhr-University Bochum, St. Josef Hospital, Bochum, Germany; 10 Institute of Human Genetics, University Bonn, Bonn, Germany; 11 Max-Planck-Institute for Neurological Research, Cologne, Germany; 12 C. and O. Vogt Institute for Brain Research, Medical Faculty, Heinrich Heine University Düsseldorf, Düsseldorf, Germany; 13 Center for Rare Diseases Bonn (ZSEB), University Hospital Bonn, Bonn, Germany; 14 Center for Economics and Neuroscience, University Bonn, Bonn, Germany; Hannover Medical School, GERMANY

## Abstract

**Objectives:**

The aim of this study was to examine the natural history of brain involvement in adult-onset myotonic dystrophies type 1 and 2 (DM1, DM2).

**Methods:**

We conducted a longitudinal observational study to examine functional and structural cerebral changes in myotonic dystrophies. We enrolled 16 adult-onset DM1 patients, 16 DM2 patients, and 17 controls. At baseline and after 5.5 ± 0.4 years participants underwent neurological, neuropsychological, and 3T-brain MRI examinations using identical study protocols that included voxel-based morphometry and diffusion tensor imaging. Data were analyzed by (i) group comparisons between patients and controls at baseline and follow-up, and (ii) group comparisons using difference maps (baseline–follow-up in each participant) to focus on disease-related effects over time.

**Results:**

We found minor neuropsychological deficits with mild progression in DM1 more than DM2. Daytime sleepiness was restricted to DM1, whereas fatigue was present in both disease entities and stable over time. Comparing results of cross-sectional neuroimaging analyses at baseline and follow-up revealed an unchanged pattern of pronounced white matter alterations in DM1. There was mild additional gray matter reduction in DM1 at follow-up. In DM2, white matter reduction was of lesser extent, but there were some additional alterations at follow-up. Gray matter seemed unaffected in DM2. Intriguingly, longitudinal analyses using difference maps and comparing them between patients and controls did not reveal any significant differences of cerebral changes over time between patients and controls.

**Conclusion:**

The lack of significant disease-related progression of gray and white matter involvement over a period of five years in our cohort of DM1 and DM2 patients suggests either a rather slowly progressive process or even a stable course of cerebral changes in middle-aged adult-onset patients. Being the first longitudinal neuroimaging trial in DM1 and DM2, this study provides useful additional information regarding the natural history of brain involvement.

## Introduction

Myotonic dystrophies type 1 and 2 (DM1 and DM2) are autosomal dominantly inherited multisystemic repeat expansion disorders and represent the most common muscular dystrophies in adulthood. Brain involvement is common and associated with executive dysfunction, mental slowing, daytime sleepiness/fatigue, behavioural abnormalities and specific personality traits usually more frequent in DM1 than DM2 [1–6]. Previous cross-sectional neuroimaging studies demonstrated brain atrophy, (sub-)cortical gray matter reduction, and white matter lesions (WML) in DM1 more than DM2. Diffusion tensor imaging (DTI) showed a widespread degradation of fiber tracts even in normal-appearing white matter [7–14]. White matter exceeded gray matter changes in adult-onset DM1 and DM2 suggesting a predominant white matter disease [9, 10, 12, 14]. Recent resting state functional MRI studies in DM1 demonstrated a reduced connectivity in specific cerebral networks underlying behavioural abnormalities [4]. The functional relevance of structural brain abnormalities is still unknown, and results of previous correlation analyses between brain morphology, neuropsychological, clinical, and genetic data were highly controversial [5, 8, 9, 12, 13, 15]. MRI changes correlated with disease duration in cross-sectional analyses, and a more neurodegenerative than neurodevelopmental origin was hypothesized [10]. A recent cross-sectional study on juvenile and adult DM1 postulated a degenerative, premature aging origin of gray matter changes in contrast to a more developmental origin of white matter alterations [9]. Additionally, a recent longitudinal neuropsychological study on cognitive abilities suggests an accelerated normal aging process [16]. On several international meetings it has been claimed to clarify the extent of disease progression over time in order to establish uniform neuroimaging, neuropsychological and neuropathological biomarkers, examination protocols and outcome measures, respectively [17–19]. Longitudinal studies evaluating spatiotemporal imaging changes are essential and missing to date [20]. We initiated the first longitudinal neuroimaging study to examine functional and structural brain involvement in classical adult-onset DM1 and DM2 over a 5-year observation period.

## Materials and methods

This is an observational longitudinal single center study registered at ClinicalTrials.gov, number NCT02729597.

The local ethics committee of the University of Bonn, Germany, approved the study protocol (No. 047/07). Informed written consent was obtained from all participants.

### Subjects

All 49 study participants belonged to a larger cohort of genetically confirmed DM1 and DM2 patients and healthy controls who had been examined previously at baseline (n = 66) [10].17/66 subjects of the initial cohort were lost to follow-up. These included six DM1 patients (mean age at baseline 44.9 ± 23.2 years, disease duration 12.8 ± 6.0 years), six DM2 patients (mean age at baseline 63.1 ± 6.2 years, disease duration 12.3 ± 12.9 years), and five control subjects (mean age at baseline 48.7 ± 4.8 years). Reasons for drop out were death (one DM1 patient died from ovarial cancer, one DM2 patient died from sepsis related to suspected systemic vasculitis, and two DM2 patients died for unknown reason), severe morbidity or immobility (two DM1 patients), unknown current address (one DM1, two DM2 patients, two controls), moving away (one DM2 patient), and participant´s preference (two DM1 patients, three controls).

For follow-up, we examined 16 adult-onset DM1, 16 DM2 patients, and 17 healthy controls 5.5 ± 0.4 years after their first visit. All baseline clinical and imaging data of these participants were recalculated excluding data of dropped-out participants, and we compared baseline with follow-up data. To assess disease severity, we used the Muscular Impairment Rating Scale (MIRS) in DM1 patients [21]. To date, there exists no equivalent scale for DM2 patients. However, all DM2 patients had proximal muscular weakness and/or myotonia.

DM1 patients lost to follow-up did not differ from DM1 patients included in our longitudinal study regarding age (*t*-test, *p* = 0.704) or disease duration (*p* = 0.854). DM2 patients lost to follow-up were significantly older (*p* = 0.001) than DM2 patients included in our longitudinal study, while disease duration was not significantly different (*p* = 0.905). Healthy control subjects lost to follow-up did not differ with respect to age from those included in our longitudinal study (*p* = 0.692).

None of our patients or controls had a past medical history of ischemic cerebrovascular disease, head trauma, brain tumor, or other central nervous system disorders. None of our patients was treated with modafinil or mexiletine. Data on vascular risk factors were gathered from each participant’s medical history and by personal interviews at follow-up visits.

In our DM1 cohort, four patients were current smokers, one patient was former smoker, two patients had diabetes mellitus, and one patient had elevated blood pressure.

In our DM2 cohort, four patients were current smokers, one patient was former smoker, three patients had elevated blood pressure, and eight patients had hyperlipidemia.

In our control cohort, one person was current smoker, six participants were former smokers, six participants had elevated blood pressure, two had diabetes mellitus, and five had hyperlipidemia.

In total, one DM1 patient, five DM2 patients, and six controls had two or more vascular risk factors.

All examinations were performed at the Department of Neurology, University Hospital Bonn, and the Life & Brain Center, Bonn, Germany, applying the identical study protocols and the identical MRI hardware and software at baseline and follow-up [10]. The local ethics committee approved the study protocol. Informed written consent was obtained from all participants.

### Neuropsychological testing

Cognitive functioning, daytime sleepiness, fatigue and depression were assessed at baseline and follow-up using a comprehensive neuropsychological test battery and various questionnaires that also had been employed in our previous study [10]. We examined verbal and figural memory using a computerized neuropsychological screening test (NeuroCogFX [22]). The Boston Naming Test was applied to evaluate semantic memory [23]. Reaction and processing speed were assessed with a symbol-counting task (subtest 1 of the Cerebraler Insuffizienztest (c.I.T.), c.I.T.S [24]), the Trail-Making Test, TMT [25], a reaction time task and the selective attention task of NeuroCogFX [22], including a simple condition as well as a choice condition/choice reaction time (subtest 1 and 2 of NeuroCogFX). For analysing executive functioning, we implemented two different interference tasks; a subtest response inhibition of the c.I.T. (c.I.T.I.) [24], and inverted choice reaction with reversed conditions of the choice reaction task (NeuroCogFX [22]). Visuoconstructive abilities were investigated using the Block design Test (part of Hamburg-Wechsler Intelligenztest für Erwachsene-Revision, HAWIE-R) [26]. We examined phonemic verbal word fluency (oral word-fluency test, subtest 6 of the Leistungsprüfsystem [27]), and semantic word fluency [28]. If available, parallel forms were applied at follow-up (e.g. verbal and figural memory recognition tasks of the computerized neuropsychological tests—NeuroCogFX).

The Beck Depression Inventory, BDI, original version, was used to assess depressive symptoms [29]. The evaluation corresponds to the German national S3-guideline for unipolar depression [30].

We applied several questionnaires to assess sleep quality, increased daytime sleepiness, fatigue, and signs of narcolepsy: Pittsburgh Sleep Quality Index, PSQI [31], Epworth Sleepiness Scale, ESS [32], Daytime Sleepiness Scale, DSS [33], Krupp's Fatigue Severity Scale, KFSS [34], and Ullanlinna-Narcolepsy Scale, UNS [35].

For further details we refer to the Supplementary Material of our previous study [10].

### Statistical analysis of clinical and neuropsychological data

We analyzed clinical and neuropsychological data using SPSS Statistics 21 (SPSS Inc., Chicago IL, USA). Analysis of covariance (ANCOVA) was performed separately for baseline and follow-up data comparing each patient group with our control group using the bimanual task of the Pegboard Puzzle [36] and age at follow-up as covariates to correct for motor impairment and age effects. BDI, sleepiness and fatigue questionnaires were compared between groups by analysis of variance (ANOVA). For longitudinal analysis of neuropsychological performance and fatigue/sleepiness questionnaires, we used two-way repeated measures analysis of variance (rANCOVA / rANOVA) to show significant differences in changes between groups over time. For longitudinal analysis of neuropsychological performance, the difference between baseline and follow-up performance in the bimanual task of the Pegboard Puzzle was used as covariate to correct for decline in motor impairment, and age at follow-up was used as covariate to correct for age effects. The neuropsychological test battery comprised several neuropsychological tests with different variables partially addressing the same cognitive domains. Therefore, the test battery was split into domains (memory, reaction, executive functioning, fluency), and the *p*-values for group comparisons were Bonferroni-corrected to adjust for multiple testing across the domains. Additionally, Cohen’s *d*–values for the effect sizes are provided.

### Brain MRI

Apart from assessing white matter lesions (WML) in conventional brain MRI scans, we applied voxel-based morphometry (VBM) and diffusion tensor imaging (DTI) techniques to analyze brain structure in more detail. VBM is a technique to quantify gray or white matter volumes/densities obtained from T1-weighted sequences using an automated and unbiased voxel-based approach. The method DTI is based on diffusion-weighted sequences and focusses on white matter microstructure via analysis of diffusivity indices that reflect tissue-specific properties.

MRI data were acquired using a 3T scanner (Magnetom Trio, Siemens) and an eight-channel head coil for signal reception. All subjects underwent the same imaging protocol as described earlier [10], enabling VBM and DTI analyses. We obtained T1-weighted 3D images using an MP-RAGE sequence with 160 slices (TR = 1300 ms, TI = 650 ms, TE = 3.97 ms, resolution 1.0×1.0×1.0 mm, flip angle 10°) and the T2-weighted 3D dataset with 192 slices (RARE; TR = 2 s, TE = 355 ms, resolution 1.0×1.0×1.0 mm, flip angle 180°). Diffusion-weighted data were acquired using single shot, dual echo, spin-echo echo planar imaging (EPI) (TR = 12 s, TE = 100 ms, 72 axial slices, resolution 1.72×1.72×1.7 mm, no cardiac gating). As parallel imaging scheme, a GRAPPA technique (acceleration factor 2.0) was chosen. Diffusion weighting was isotropically distributed along 60 directions (b-value = 1000 s/mm^2^). Additionally, seven data sets with no diffusion weighting were acquired initially and interleaved after each block of 10 diffusion weighted images as anatomical reference for motion correction. The high angular resolution of the diffusion weighting directions improves the robustness of probability density estimation by increasing the signal-to-noise ratio and reducing directional bias. Image quality was controlled by visual inspection, and images with artefacts were excluded from further data analysis.

#### Grading of white matter lesions

WML were quantified on baseline and follow-up T2-weighted images according to the age-related white matter change score (ARWMC) [37]. The grading within five regions, separately for each hemisphere, ranged from 0 (no lesions) to 3 (diffuse involvement of the entire region).

#### Voxel-based morphometry analyses

After quality check, three DM1 patients, one DM2 patient, and four controls were excluded from VBM analyses due to motion artefacts. VBM analysis was performed in 13 DM1 patients (nine females; mean age 48.5 ± 6.7 years at follow-up), 15 DM2 patients (six females; mean age 54.1 ± 8.4 years at follow-up), and 13 controls (seven females; mean age 54.6 ± 9.6 years at follow-up).

Image processing and statistical analyses were carried out according to the optimized VBM protocol using MATLAB 7.7.0 and statistical parametric mapping (SPM 5; http://www.fil.ion.ucl.ac.uk/spm/software/spm5) [38]. For details on VBM data analysis we refer to Minnerop *et al*., 2011 [10]. For normalisation and segmentation of T1-weighted MRI data, default settings of the VBM toolbox VBM 5.1 (http://dbm.neuro.uni-jena.de/) were applied [39, 40]. As we expected effects of relative volumes, we used modulated images to correct for different brain sizes. The resulting normalised, segmented and modulated images were resampled to a voxel size of 1.0 x 1.0 x 1.0 mm and smoothed with a 10-mm Gaussian kernel. The latter was chosen in accordance with the recommendation for the smoothing of modulated images included in the VBM toolbox VBM 5.1. Statistical comparisons between patient groups and controls were performed separately for gray and white matter at baseline and follow-up. For the analysis of longitudinal changes we did not use paired *t*-tests within each group as this approach lacks the comparison with healthy controls and would not allow disentangling merely aging-related from disease-related changes. To focus the analysis on disease-related longitudinal changes over time, we therefore applied a different approach. We created difference maps with the ImCalc function of SPM by subtracting gray and white matter maps at follow-up from baseline (map_baseline_−map_follow-up_), enabling group comparison of these difference maps between patient groups and healthy controls. To further consider group-specific aging, ANCOVAs with mean-centered group-specific age as covariate were performed in all cross-sectional and longitudinal analyses. Since the impact of aging is in particular relevant for analysing disease progression over time, additional longitudinal analyses were conducted with age at follow-up as single regressor across all groups to model general effects of age on disease progression. The results of group analyses were explored at a false discovery rate-corrected threshold of *p* < 0.05 at voxel-level with an extended cluster threshold of 10 voxels.

#### Diffusion tensor imaging analyses

After quality check, one DM1 patient and three controls were excluded from DTI analyses on account of extensive extinction artifacts. DTI analysis was performed in 15 DM1 patients (nine females; mean age 48.1 ± 6.7 years at follow-up), 16 DM2 patients (seven females; mean age 53.8 ± 8.2 years at follow-up), and 14 controls (eight females; mean age 55.2 ± 9.5 years at follow-up). Due to extinction artifacts in the cerebellum that were frequently observed at follow-up, statistical analyses of the cerebellar region were omitted at follow-up and in over-time analyses of difference images.

For details on DTI data analysis we refer to Minnerop *et al*., 2011 [10]. Preprocessing and analysis of diffusion data were done with an in-house protocol using FMRIB software library (FSL) 4.1.3 tools (available at www.fmrib.ox.ac.uk/fsl). DICOM images were converted into a 4D Nifti-File. Motion correction was then applied on all images using 7-parameter global rescale registration with a mutual information cost function and tri-linear interpolation as implemented in FLIRT, FMRIB Linear Image Registration Tool [41]. Baseline b0 images were aligned to a reference b0 image and the resulting linear transformation matrixes were then applied to the diffusion weighted images following each baseline b0 image. A binary mask differentiating between brain and skull structures was calculated for brain extraction using BET, Brain Extraction Tool, and applied to all images. Next, the following indices were generated using the DTIfit algorithm [42]: fractional anisotropy (FA), mean diffusivity (MD), eigenvalues (λ_1_, λ_2_, λ_3_) of the diffusion tensor with axial diffusivity (AD, λ_1_) presumed to be the diffusivity parallel/along the axon, and radial diffusivity RD [(λ_2_+λ_3_)/2] presumed to be the diffusivity perpendicular to the axon. Voxelwise analysis of the resulting maps was carried out using the tract-based spatial statistics (version 1.1) tool also included in FSL [43]. Group comparisons at baseline and follow-up between patient groups and controls were performed with respect to fractional anisotropy (FA), mean (MD), axial (AD), and radial diffusivity (RD). As mentioned already in the VBM methods description, we intended to focus the analysis on disease-related longitudinal changes over time. Therefore, skeletonised difference maps for each subject (e.g.: FA-skeletonised _follow-up_−FA-skeletonised _baseline_) were created with FSL tools, enabling group comparison of these differences maps between patient group and healthy controls. To further consider group-specific aging, ANCOVAs with mean-centred group-specific age as covariate were performed in all analyses (with respect to FA, MD, AD, RD). Since the impact of aging is in particular relevant for analysing disease progression over time, additional longitudinal analyses were conducted with age at follow-up as single regressor across all groups to model general age effects. The results of group analyses were explored at a family wise error-corrected threshold of *p* < 0.05.

We created tract-specific masks separately for each group (DM1, DM2, controls) by using FSLUTILS tools that included only those voxels with reduced FA in DM patients in comparison to controls at baseline in our ANCOVAs. Using these masks, region-specific mean FA values were calculated at baseline and at follow-up (S1 Table).

## Results

### Clinical characteristics

Cardiovascular risk factors were not more frequent in both patient cohorts compared to our controls. DM1 patients were younger than controls (*p* = 0.009). At baseline and follow-up, DM1 patients had poorer motor performance than controls in the bimanual pegboard task (baseline *p* = 0.041, follow-up *p* = 0.018). DM1 patients showed a significant worsening of muscular strength (Muscular Impairment Rating Scale (MIRS), *p* = 0.006) and fine motor skills over time (as did controls; bimanual pegboard task, DM1 *p* = 0.003, controls *p =* 0.022). At baseline and follow-up, DM1 (baseline *p* = 0.001, follow-up *p* < 0.001) and DM2 patients (baseline *p* = 0.002, follow-up *p* < 0.001) had more depressive symptoms than controls. The number of patients with clinically relevant depressive symptoms remained stable over time in DM1 and mildly increased in DM2. Statistical analyses did not show significant changes of depressive symptoms in both disorders and controls over time (Table 1).

**Table 1:** Clinical characteristics of DM1 and DM2 patients and controls at baseline (T1) and follow-up (T2).

	DM1		DM2		Controls	
	T1	T2	T1	T2	T1	T2
*n* *Sex (M/F)*	16 6/10		16 9/7		17 9/8	
Age, given in years	**42.5 ± 6.5** (30.8–56.0)	**48.2 ± 6.5** (36.7–61.9)	48.5 ± 8.4 (38.4–64.6)	53.8 ± 8.3 (43.4–69.6)	50.5 ± 9.8 (33.5–67.9)	56.2 ± 9.7 (39.4–72.9)
Time interval, given in years	5.8 ± 0.3		5.2 ± 0.5		5.7 ± 0.2	
CTG repeat length	618.1 ± 284.1 (180–1200)		**-**		**-**	
Educational level, sum score	11.3 ± 1.8 (8–14)		11.3 ± 2.4 (8–14)		12.1 ± 1.5 (9–14)	
Duration of disease, given in years	13.4 ± 7.5 (2–25)	19.1 ± 7.6 (7–31)	11.4 ± 9.1 (1–36)	16.9 ± 9.1 (6–41)	**-**	**-**
Severity of disease, Muscular Impairment Rating Scale	3.4 ± 1.0 (2–5)	3.9 ± 0.6 (3–5)	**-**	**-**	**-**	**-**
Fine motor skills, Purdue Pegboard bim., cut off <11 pairs in 30s	**10 ± 2.5** (5–14)	**8.4 ± 3.6** (2–13)	10.6 ± 2.1 (7–13)	10.0 ± 1.6 (7–13)	11.5 ± 1.3 (9–14)	10.9 ± 1.7 (7–13)
BDI Score	**7.8 ± 5.1** (1–20)	**9.4 ± 4.3** (2–18)	**8.5 ± 6.7** (0–24)	**10.9 ± 7.6** (1–26)	1.9 ± 3.5 (0–13)	3.0 ± 2.9 (0–9)
Depression, absolute number of participants with a BDI score above cut off (>10)	5	5	6	7	1	0

Given values are mean ± SD (otherwise indicated differently), range values are given in brackets.

T1 = baseline examination, T2 = follow-up examination, M = male, F = female

Educational levels were assessed as a combination score of graduation and professional qualification (sum score).

Purdue Pegboard bimanual: lower values correspond to a worse test result.

BDI: Beck Depression Inventory, original version, to assess depressive symptoms (BDI sum score <10: normal or clinically inapparent, 10–19: mild depression, 20–29: moderate depression, >29: severe depression) [30].

Bold = significant difference compared with control group, *p* < 0.05

### Neuropsychological performance, sleepiness and fatigue at baseline, follow-up, and over time

DM1 and DM2 patients showed significantly higher susceptibility to interference (c.I.T.I.) at baseline and follow-up compared with our control group. At baseline, DM2 patients showed poorer performance in focused attention (c.I.T.S.) compared with controls. Significant results were associated with high effect sizes (Table 2).

**Table 2:** Neuropsychological performance controlled for motor function and age effects in DM1 and DM2 patients and healthy controls, test results at baseline (T1) and follow-up (T2).

Domains/ Tests		DM1 (n = 16)	DM2 (n = 16)	Control group (n = 17)	DM1 compared with controls	DM2 compared with controls
**Memory**						
Naming (Boston Naming), a	T1	55.50 ± 3.60	52.88 ± 8.31	57.29 ± 3.16	0.26 (0.530)	3.25 (0.71)
	T2	55.31 ± 5.13	55.75 ± 3.61	57.06 ± 4.31 [n = 16]	0.04 (0.370)	0.70 (0.329)
Verbal memory recognition (NeurocogFX), a	T1	44.09 ± 2.56	39.91 ± 7.78	40.91 ± 3.10	6.49 (-1.115) ***p* = 0.048** !	0.05 (0.171)
	T2	40.53 ± 11.28	42.44 ± 3.86	38.18 ± 11.16	0.39 (-0.237)	0.53 (-0.504)
Figural memory recognition (NeurocogFX), a	T1	6.25 ± 5.65	8.06 ± 9.23	8.00 ± 6.34	0.27 (0.291)	0.66 (-0.008)
	T2	5.69 ± 6.94	11.31 ± 6.57	9.65 ± 6.39	0.01 (0.594)	0.30 (-0.256)
**Reaction**						
Focused attention (c.I.T.S.), b	T1	18.00 ± 4.10	20.19 ± 5.37	15.94 ± 3.05	0.53 (-0.573)	7.16 (-0.981) ***p* = 0.048**
	T2	20.88 ± 4.60	20.56 ± 5.06	17.35 ± 2.91	3.54 (-0.924)	4.99 (-0.784)
Psychomotoric speed (TMT A), b	T1	31.69 ± 10.36	35.06 ± 14.73	34.53 ± 14.81	0.27 (0.221)	0.04 (-0.036)
	T2	38.44 ± 12.15	32.63 ± 12.82	37.06 ± 10.91	0.01 (-0.12)	1.06 (0.373)
Reaction time (NeurocogFX), b	T1	262.06 ± 63.53	279.00 ± 73.22	258.06 ± 45.21	0.48 (-0.073)	0.41 (-0.347)
	T2	288.50 ± 33.62	291.88 ± 38.72	264.35 ± 37.29	2.01 (-0.679)	2.71 (-0.725)
Selective attention (Choice reaction time, NeurocogFX), b	T1	88.00 ± 33.03	143.19 ± 59.52	135.06 ± 50.66	6.04 (1.093)	0.83 (-0.147)
	T2	119.94 ± 78.22	107.31 ± 94.10	100.18 ± 29.9	0.81 (-0.338)	0.17 (-0.103)
**Executive functioning**						
Interference (c.I.T.I), b	T1	25.63 ± 5.76	25.06 ± 5.01	18.65 ± 3.22	**7.24 (-1.509)** ***p* = 0.048**	**13.88 (-1.532)** ***p* = 0.004**
	T2	27.50 ± 6.06	25.63 ± 5.60	19.00 ± 2.55	**9.56 (-1.849)** ***p* = 0.016**	**17.69 (-1.54)** ***p* = < 0.001**
Interference (NeurocogFX), b	T1	388.06 ± 55.44	436.50 ± 84.57	415.18 ± 77.86	1.19 (0.399)	0.38 (-0.263)
	T2	409.94 ± 47.43	422.88 ± 64.36	418.71 ± 83.77	0.34 (0.128)	0.56 (-0.063)
Attention shift, mental flexibility (TMT B), b	T1	96.56 ± 51.37	82.94 ± 28.84	82.59 ± 32.80	0.49 (-0.326)	0.25 (-0.011)
	T2	121.44 ± 76.61	90.31 ± 48.37	91.35 ± 39.22	0.07 (-0.499)	0.00 (0.024)
Visual-spatial / visual-constructive abilities (Block design), a	T1	25.25 ± 10.38	33.06 ± 7.77	28.94 ± 7.50	1.03 (0.41)	1.98 (-0.54)
	T2	23.69 ± 11.22	28.56 ± 10.20	31.41 ± 8.27	2.30 (0.787)	0.86 (0.308)
**Fluency**						
Phonematic fluency, a	T1	31.25 ± 6.79	30.19 ± 8.85	34.18 ± 9.00	0.25 (0.366)	0.21 (0.447)
	T2	32.19 ± 7.01	31.81 ± 10.18	36.29 ± 8.92	0.01 (0.509)	0.48 (0.469)
Semantic fluency, a	T1	23.94 ± 5.88	22.81 ± 6.26	24.35 ± 3.92	1.21 (0.083)	0.07 (0.297)
	T2	24.31 ± 6.03	23.63 ± 5.05	25.12 ± 6.91	0.93 (0.125)	0.47 (0.245)

The neuropsychological test battery included 13 tests, analysing memory, reaction, executive functioning, and fluency in DM1 and DM2 patients and controls at baseline (T1) and follow-up (T2).

Given values in columns 3 to 5 are mean absolute test results ± SD at baseline and follow-up. Lower *n* due to a missing value for a single score is given in squared brackets. Given values in columns 6 and 7 are *F*-values of group comparisons; Cohen´s *d*-values for the effect sizes are given in brackets. Bimanual Pegboard task and age were used as covariates to correct for motor impairment and age effects.

Bold = significant difference compared with control group, Bonferroni corrected; *p* < 0.05.

a = higher number indicating better results

b = lower number indicating better results

! = patients performed better than controls.

**Fig 1:**
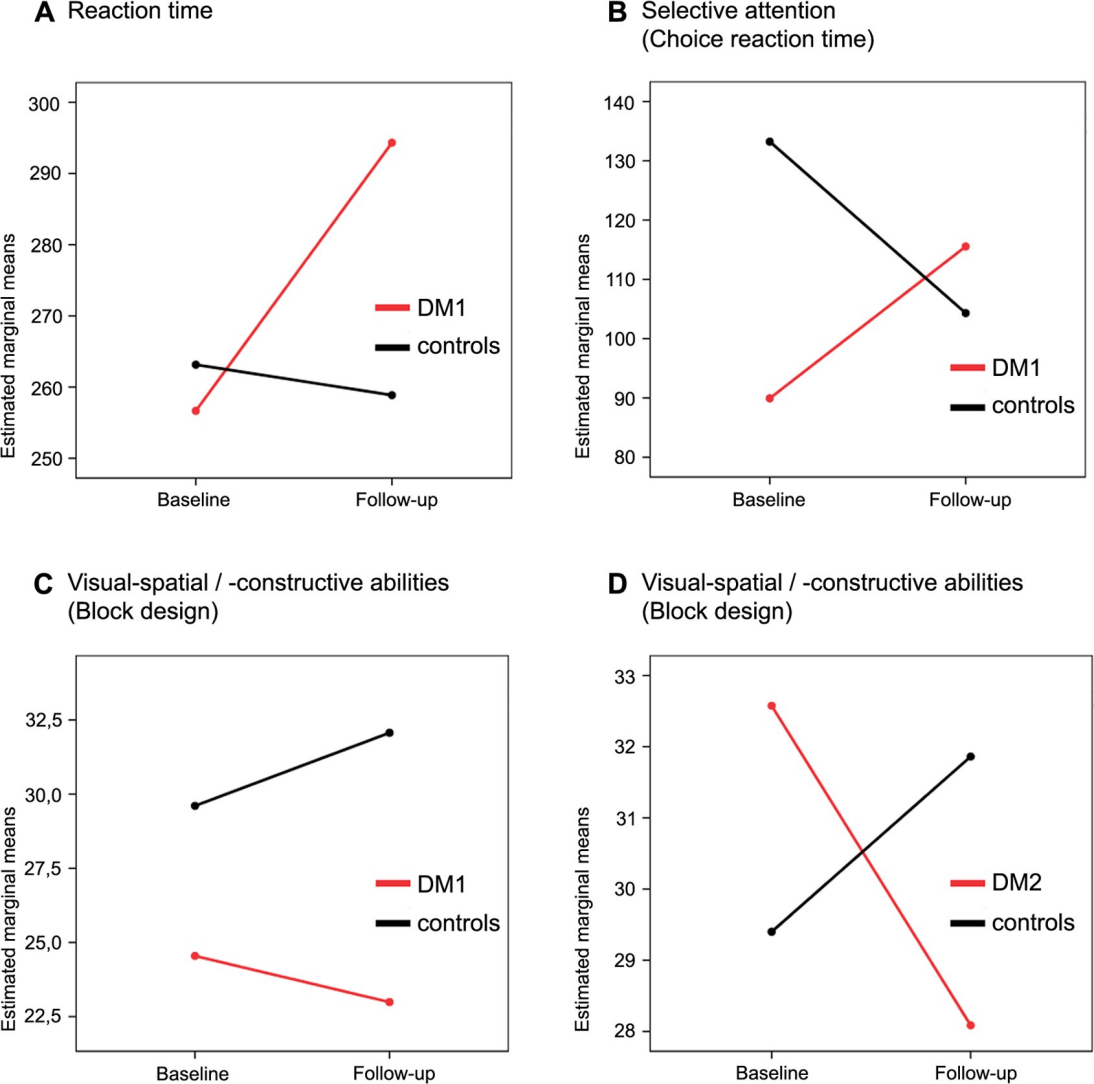
Differences in changes of neuropsychological test performances between groups over time. (A, B, C) Comparison of DM1 patients and controls over time. (A) *Reaction time*: indicating a decline in DM1 patients compared to controls (*p = 0*.*047*, *difference of means = 14*.*49*, *95% confidence interval (CI)*: *-21*.*53 to 50*.*51*). (B) *Choice reaction time test*: indicating a decline in selective attention in DM1 patients over time compared to controls (*p = 0*.*039*, *difference of means = -16*.*03*, *95% CI*:*-54*.*90 to 22*.*84*). (C) *Block design*: indicating a decline in DM1 patients over time compared to controls (*p = 0*.*048*, *difference of means = -7*.*07*, *95% CI*: *-15*.*23 to 1*.*09*). (D) Comparison of DM2 patients and controls over time, *Block design*: indicating a decline in DM2 patients over time compared to controls (*p = 0*.*003*, *difference of means = -0*.*30*, *95% CI*: *-5*.*31 to 4*.*71*). The bimanual *Pegboard task* and age at follow-up were used as covariates to correct for motor impairment and age effects. *Block design* = higher numbers indicating better results *Choice reaction time test*, *Reaction time* = lower numbers indicating better results Estimated marginal means describe mean values of the respective neuropsychological test (reaction time, choice reaction time in selective attention test, score in block design test) that are adjusted for other variables (age, motor performance).

rANCOVA analysis showed a significant difference in changes over time between DM1 patients and controls in the *Reaction time* task, in the *Choice reaction time test* probing selective attention and in the *Block design* testing visual-spatial and visual-constructive abilities. In DM2 patients, rANCOVA revealed a significant difference in changes over time in *Block design* only. In contrast to controls who improved in the *Block design*, *Reaction time* and *Choice reaction time test*, patients showed a decline (Fig 1).

At baseline and follow-up, the analysis of sleep quality, daytime sleepiness and fatigue questionnaires showed significantly higher scores–indicating more symptoms—in all scales in DM1 compared to controls and in 2/5 questionnaires indicating sleep quality and fatigue in DM2 (Table 3). Comparing each patient group with controls by rANOVA did not reveal any significant differences in changes over time in sleepiness and fatigue scales between groups.

**Table 3:** Sleep and fatigue scales in DM1 and DM2 patients compared with healthy controls at baseline and follow-up.

	Cut-off values		DM1 (n = 16)	DM2 (n = 16)	Control group (n = 17)	DM1 compared with controls	DM2 compared with controls
**PSQI total score**	>5	T1	**5.2** ± 2.27 6/15 (40.0%)	**6.40** ± 3.62 8/15 (53.3%)	3.00 ± 2.07 1/16 (6.3%)	**7.97** (1.014) ***p* = 0**.**009**	**10.48** (1.282) ***p* = 0**.**003**
		T2	**5.87** ± 3.07 6/16 (37.5%)	**5.31** ± 4.08 8/16 (50.0%)	2.53 ± 2.42 2/17 (11.8%)	**12.12** (1.213) ***p* = 0.002**	**5.76** (0.835) ***p* = 0**.**023**
**KFSS**	>3.7	T1	**4.05** ± 1.73 9/16 (56.3%)	**4.49** ± 1.71 11/16 (68.8%)	2.26 ± 0.79 0/16 (0%)	**14.08** (1.361) ***p* = 0**.**001**	**22.33 (**1.674) ***p* < 0**.**001**
		T2	**4.30** ± 1.46 11/16 (68.8%)	**4.25** ± 1.66 9/16 (56.3%)	1.94 ± 0.92 2/17 (11.8%)	**31.13** (1.948) ***p* < 0**.**001**	**24.61** (1.736) ***p* < 0**.**001**
**DSS**	>10	T1	**10.63** ± 3.07 8/16 (50.0%)	8.00 ± 4.62 6/16 (37.5%)	7.06 ± 3.17 3/16 (18.8%)	**10.41** (1.144) ***p* = 0**.**003**	0.45 (0.237)
		T2	**12.25** ± 5.13 8/16 (50.0%)	8.06 ± 4.06 4/16 (25.5%)	6.00 ± 4.26 1/16 (6.3%)	**14.06** (1.326) ***p* = 0**.**001**	1.97 (0.495)
**ESS**	>10	T1	9.00 ± 3.25 6/16 (37.5%)	7.19 ± 3.76 2/16(12.5%)	6.27 ± 3.08 2/15 (13.3%)	**5.77** (0.861) ***p* = 0**.**023**	0.55 (0.267)
		T2	**10.62** ± 4.19 9/16 (56.3%)	6.75 ± 3.53 2/16(12.5%)	5.76 ± 3.05 1/17 (5.9%)	**14.62** (1.326) ***p* = 0**.**001**	0.74 (0.338)
**UNS**	>13	T1	8.94 ± 3.71 2/16 (12.5%)	5.31 ± 2.41 0/16 (0%)	6.00 ± 2.19 0/16 (0%)	**7.42** (0.965) ***p* = 0**.**011**	0.71 (0.3)
		T2	9.94 ± 5.58 4/16 (25.0%)	6.44 ± 2.76 0/16 (0%)	5.82 ± 2.65 0/17 (0%)	**7.46** (0.953) ***p* = 0**.**010**	0.43 (0.229)

Score values of sleep and fatigue scales at baseline (T1) and follow-up (T2), mean ± SD are given in columns 4 to 6. Mean values that score above the clinical cut-off of pathological performing are indicated in bold. Numbers of participants with scores above the clinical cut-off of pathological performing (percentages of patients scoring above cut-off values) are also given in columns 4 to 6, and lower *n* due to missing values are indicated. Results of group comparisons are given in columns 7 to 8 (F-values; significant differences, *p* < 0.05, are indicated in bold). Cohen´s *d*-values for the effect sizes are given in brackets.

### White matter lesions at baseline, follow-up, and over time

The mean total ARWMC score significantly differed from controls at baseline in DM1. Subscores for frontal brain regions significantly differed from controls in DM1 at baseline and follow-up. Temporal WML were restricted to DM1. There were no significant differences in the presence and extent of WML between DM2 and controls and between baseline and follow-up within each patient group and controls (Table 4).

**Table 4:** White matter hyperintensities rated according to the ARWMC scale in DM1 and DM2 patients and control subjects at baseline (T1) and follow-up (T2).

	DM1		DM2		Control group	
	T1	T2	T1	T2	T1	T2
**Total Score**	2.25 ± 3.00	2.44 ± 3.12	0.75 ± 1.53	0.88 ± 1.78	0.67 ± 0.91	0.94 ± 1.26
*p*-value (compared to controls)	0.04*	0.07	0.85	0.90		
**Frontal subscore**	1.19 ± 1.60	1.25 ± 1.57	0.44 ± 0.85	0.50 ± 1.03	0.11 ± 0.47	0.17 ± 0.71
*p*-value (compared to controls)	0.01*	0.01*	0.16	0.28		
**Parieto-occipital subscore**	0.69 ± 1.30	0.75 ± 1.39	0.31 ± 0.87	0.38 ± 0.89	0.5 ± 0.71	0.67 ± 0.97
*p*-value (compared to controls)	0.60	0.84	0.49	0.37		
**Temporal subscore**	0.38 ± 1.09	0.38 ± 1.09	0	0	0	0
*p*-value (compared to controls)	0.16	0.15				
**Infratentorial subscore**	0.00	0.06 ± 0.25	0.00	0.00	0.06 ± 0.24	0.11 ± 0.32
*p*-value (compared to controls)	0.34	0.63	0.34	0.18		

Given values are mean ± SD.

*p < 0.05; significant differences between patient and control groups.

### Voxel-based morphometry analyses at baseline, follow-up, and over time

**Fig 2:**
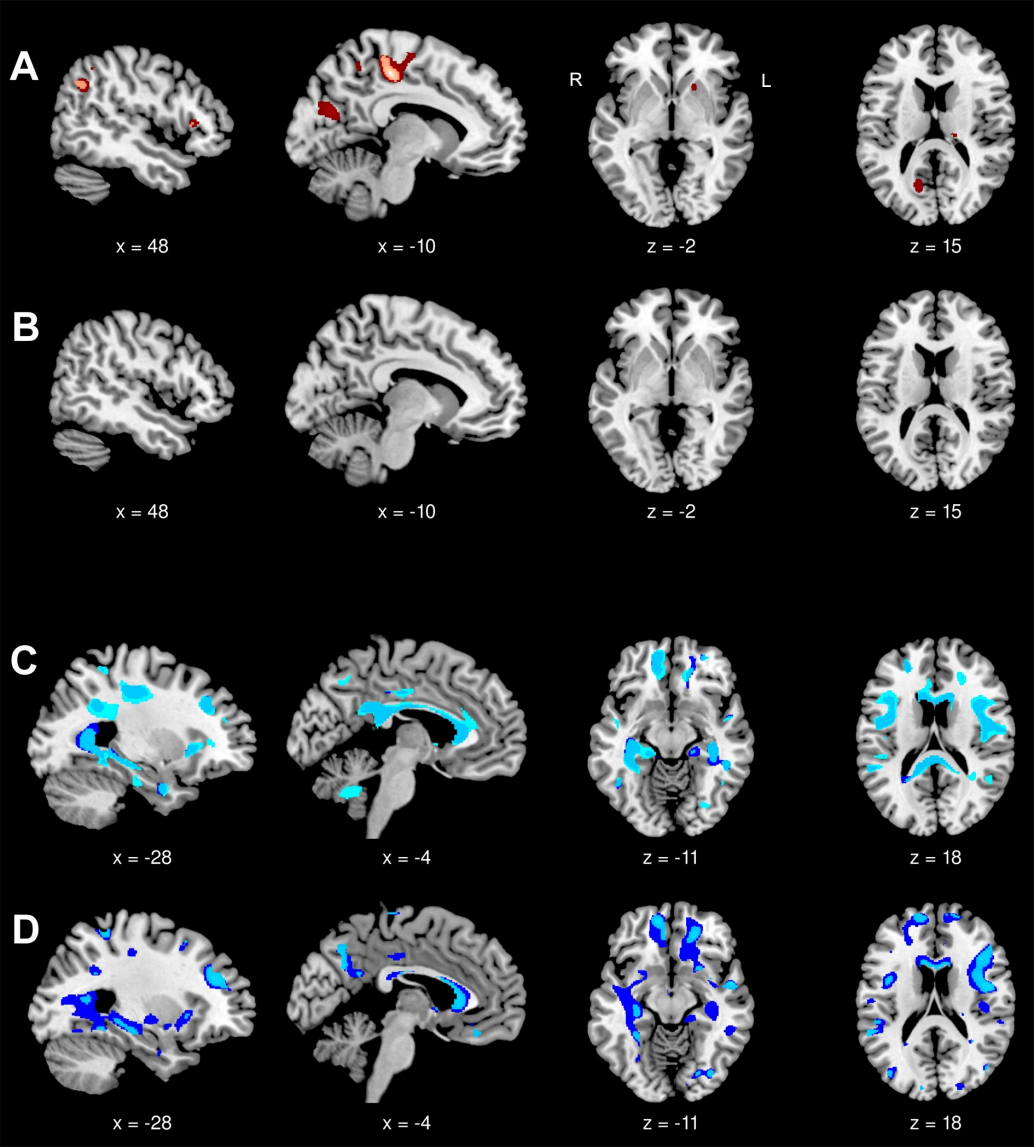
Voxel-based morphometry, group comparisons at baseline and follow-up. Displayed results of VBM analyses are based on a threshold of *p*_false discovery rate_ < 0.05 at voxel-level with an extended cluster threshold of 10 voxels. The coordinates refer to the MNI reference space. VBM analyses were performed in 13 DM1, 15 DM2 patients, and 13 controls. (A) Gray matter decrease in DM1 patients compared with controls at baseline (light red) and at follow-up (light and dark red). Dark red areas had not been affected at baseline. (B) Gray matter decrease in DM2 patients compared with controls at baseline and at follow-up (no clusters detected). (C) White matter decrease in DM1 patients compared with controls at baseline (light blue) and at follow-up (light and dark blue). (D) White matter decrease in DM2 patients compared with controls at baseline (light blue) and at follow-up (light and dark blue). Dark blue areas had not been affected at baseline.

Mild gray matter reduction was present in DM1 compared to controls at baseline and more pronounced at follow-up. At baseline, it was located in the left middle cingulum, paracentral lobules, right angular gyrus, triangular inferior frontal gyrus and supplementary motor area (SMA). At follow-up, further regions like left SMA, (pre-)cuneus and middle occipital gyrus, right precentral, and inferior parietal gyrus, right orbital middle frontal gyrus, right putamen and posterior thalamus were affected (Fig 2A). There were no gray matter reductions in DM2 patients compared to controls at baseline and at follow-up (Fig 2B). White matter reduction was ubiquitous and widespread at baseline and follow-up in both diseases. While in DM1 the pattern and extent remained almost stable between group comparisons at baseline and follow-up (Fig 2C), the decrease of white matter at follow-up exceeded that observed at baseline in DM2 (Fig 2D). However, there were no significant differences in changes over time of gray and white matter between both patient groups and controls.

### Diffusion tensor imaging analyses

#### DM1 versus controls at baseline, follow-up, and over time

**Fig 3:**
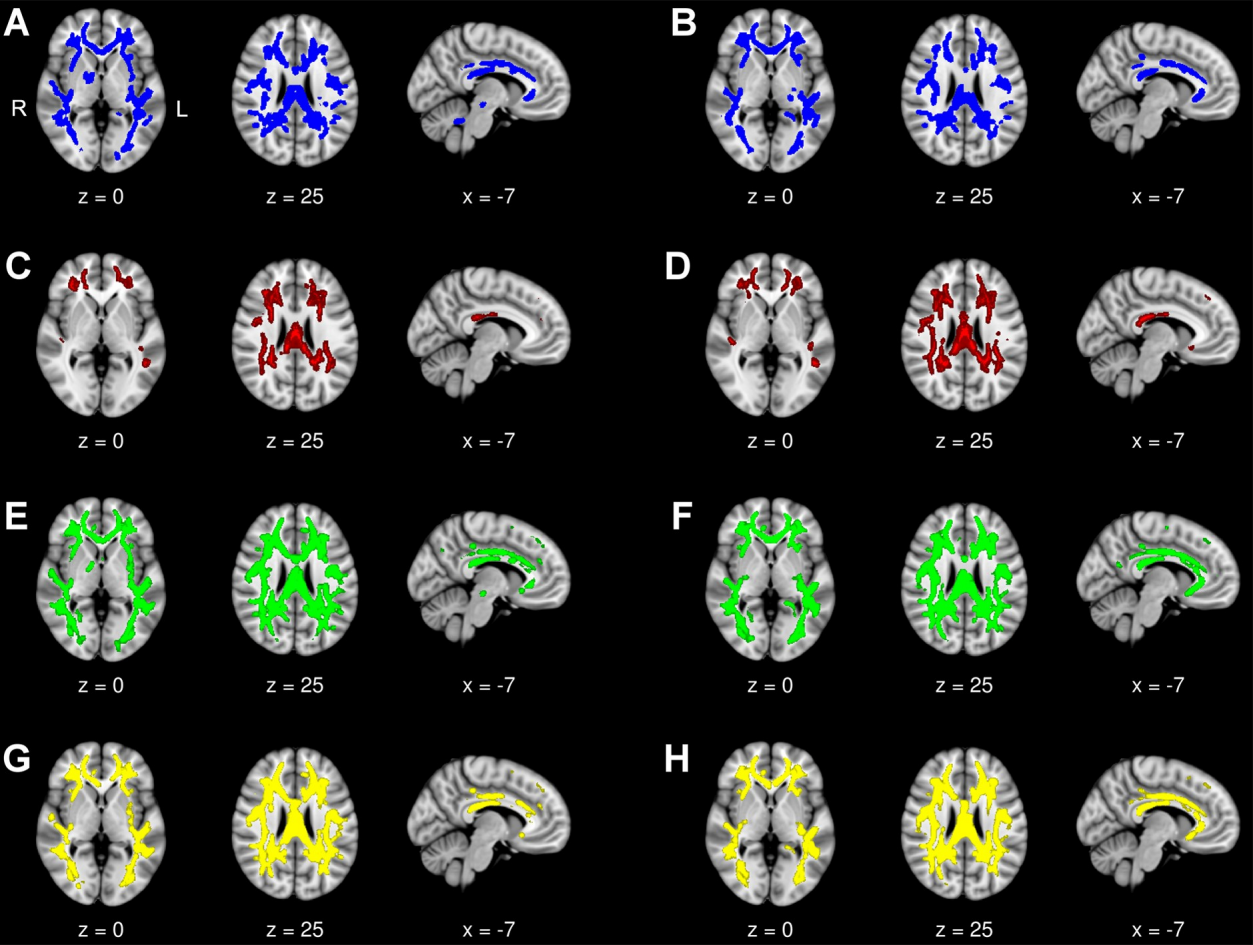
DM1 patients versus controls. **Diffusion tensor imaging group comparison at baseline and follow-up.** Displayed results of tract-based spatial statistics analyses of different diffusivity indices (FA, AD, RD, MD) are based on a corrected threshold of *p*_family wise error_ < 0.05 and overlaid on the MNI152 Template (resolution 1x1x1 mm) included in FSL. The coordinates refer to the MNI reference space. DTI analyses were performed in 15 DM1 patients and 14 controls. (A) FA reduction in DM1 patients compared with controls at baseline and (B) follow-up. (C) Increase in AD in DM1 patients compared with controls at baseline and (D) follow-up. (E) Increase in RD in DM1 patients compared with controls at baseline and (F) follow-up. (G) Increase of MD in DM1 patients compared with controls at baseline and (H) follow-up.

At baseline, we found ubiquitous decrease of white matter microstructural integrity in DM1 patients, disclosed by FA reduction mostly accompanied by increased RD and MD and partially mirrored–albeit to a less extent—by increased AD. The corpus callosum (genu, body, and splenium) and forceps minor and major were affected. FA reduction was also significant in association fibers (superior and inferior longitudinal fascicles, inferior fronto-occipital and uncinate fascicles, cingulum bundles), and in projection fibers (within both external and right internal capsules, bilateral corticospinal tracts within the parietal lobes). At follow-up, we found highly similar results compared to baseline. Nevertheless, the fornix was involved at follow-up only, and there were few regions with additional FA reduction at follow-up that were predominantly ascribed to callosal fiber tracts and peripheral parts of association fibers. FA reductions within brainstem and cerebellum, visible at baseline, could not be evaluated at follow-up due to extinction artefacts (Fig 3).

There were no differences in FA, AD, RD and MD changes between patients and controls over time in our longitudinal DTI analysis, independently if age was modelled as group-specific covariate or as group-independent single regressor. These results remained unchanged, even if the *p*-values were corrected by the threshold-free-cluster enhancement approach to increase sensitivity.

#### DM2 versus controls at baseline, follow-up, and over time

**Fig 4:**
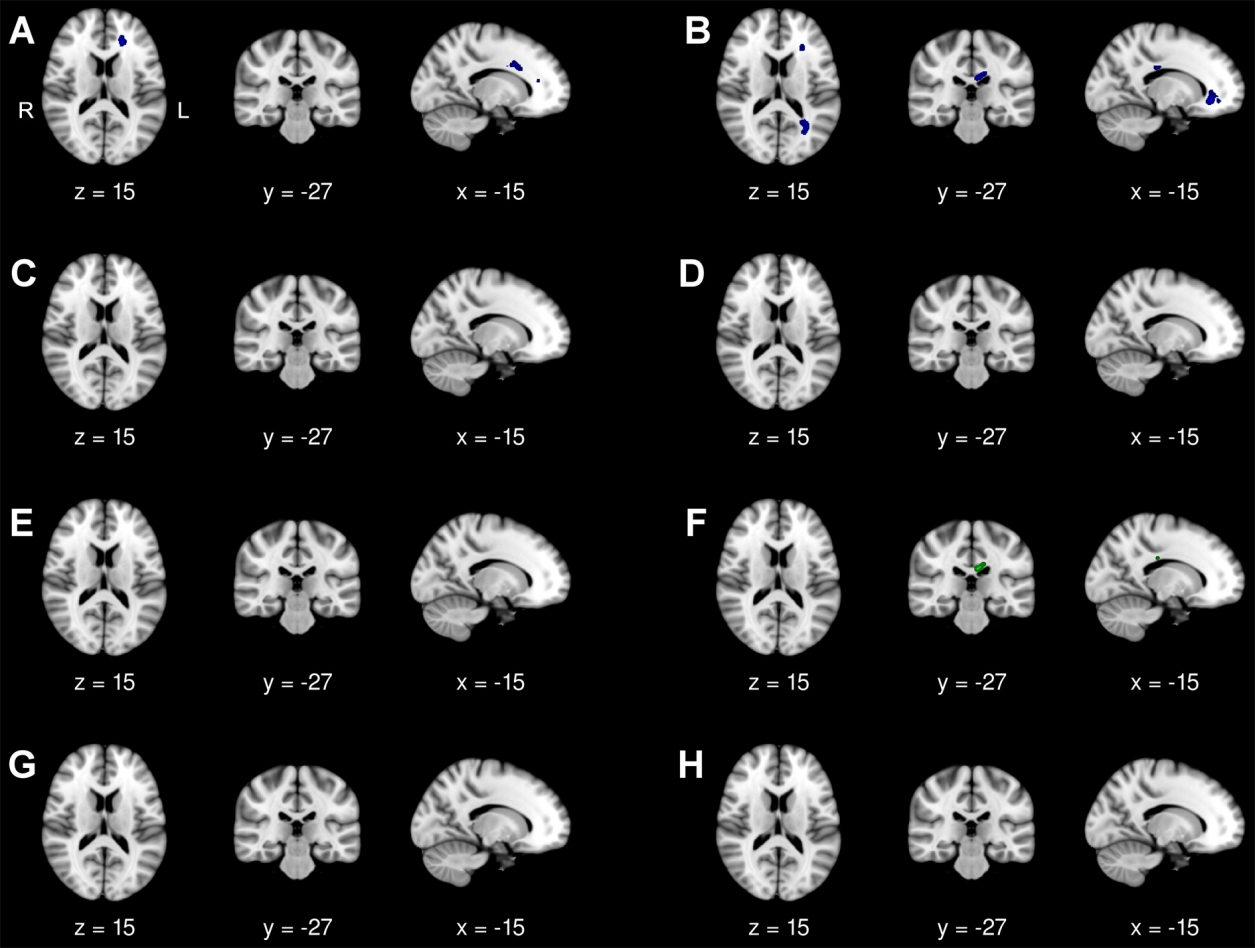
DM2 patients versus controls. **Diffusion tensor imaging group comparison at baseline and follow-up.** Displayed results of tract-based spatial statistics analyses of different diffusivity indices (FA, AD, RD, MD) are based on a corrected threshold of *p*_family wise error_ < 0.05 and overlaid on the MNI152 Template (resolution 1x1x1 mm) included in FSL. The coordinates refer to the MNI reference space. DTI analyses were performed in 16 DM2 patients and 14 controls. (A) FA reduction in DM2 patients compared with controls at baseline and (B) follow-up. (C) No increase in AD in DM2 patients compared with controls at baseline (no significant voxels detected) and (D) follow-up (no significant voxels detected). (E) No increase in RD in DM2 patients compared with controls at baseline (no significant voxels detected), but at (F) follow-up. (G) No increase of MD in DM2 patients compared with controls at baseline (no significant voxels detected) and (H) follow-up (no significant voxels detected).

At baseline, we found FA reduction in the callosal body and in parts of the left forceps minor. At follow-up, isthmus and genu of the corpus callosum, both forceps minor, the left inferior longitudinal and inferior fronto-occipital fascicles additionally showed FA reduction. There was an increase of RD within the callosal body at follow-up only, but no increase of MD or AD at baseline or follow-up (Fig 4).

In our longitudinal DTI analysis there were no differences in FA, RD, and MD changes over time between DM2 patients and controls, independently if age was modelled as group-specific covariate or as group-independent single regressor. There was a higher increase of AD over time in controls compared to DM2 patients which was localised within the genu of the corpus callosum and right forceps minor. The results remained unchanged, even if the *p*-values were corrected by the threshold-free-cluster enhancement approach to increase sensitivity.

## Discussion

We examined functional and structural brain involvement in adult-onset DM1 and DM2 over a timespan of five to six years. Particularly, we aimed to contribute longitudinal neuroimaging results to help to elucidate the still open question if cerebral changes emerge from an ongoing progressive neurodegenerative process [44].

In our previous cross-sectional baseline study we included thorough regression analyses, revealing associations of white matter changes in DM1 and DM2 with several clinical parameters (age, disease duration, repeat length (DM1), motor impairment (MIRS), scores for sleepiness, fatigue, depression, etc.), but not with neuropsychological performance. The association of white matter changes with age and disease duration further underlined the relevance of the still open question whether DM1 and DM2 are ongoing progressive neurodegenerative disorders and stressed the need for longitudinal data [10].].

Similar to some data published earlier, in our current study we found only minor neuropsychological deficits in classical adult-onset DM1 and DM2 at baseline and follow-up that predominantly affected executive functioning [1, 10, 12, 45]. When compared to controls, a few neuropsychological functions like visual spatial/constructive abilities showed a significant decline over time in DM1 and DM2 which was partly due to an improvement of controls. This might be attributed to a re-test effect in controls, not present in patients [46]. A recent study using functional MRI suggests that a balance between loss of connectivity and compensatory mechanisms in different brain networks might explain the relatively mild cognitive deficits in some DM1 patients [45]. Discrepancies between our results and previous studies showing more extensive deficits in myotonic dystrophies might partly be explained by our correction for fine motor impairment [2, 6, 7, 47]. Our results are in line with longitudinal neuropsychological studies on DM1 and DM2 that predominantly revealed a decline in executive abilities, however did not test against healthy controls [6, 48]. On the contrary, a recent longitudinal study by Gallais et al. revealed rather stable executive results, but a decline in verbal memory, attention, and psychomotor speed [16]. These inconsistencies indicate the need for more uniform neuropsychological tests and protocols in order to compare and assemble data from different centers and disease cohorts [17].

Depressive symptoms were present in DM1 and DM2 and were stable over time. The absolute number of patients with clinically relevant symptoms was higher in DM2 and mildly increased over time. This is in line with our previous observation that depressive symptoms might be more prevalent in the long-term course of DM2 compared to DM1 patients who might adapt to their symptoms earlier [10].

DM1 patients had more signs of narcolepsy as tested by the Ullanlinna Narcolepsy Scale than DM2 patients which had been demonstrated earlier [10]. Daytime sleepiness was restricted to DM1, whereas fatigue was present in DM1 and DM2. This is fully in line with previously published data suggesting the absence of excessive daytime sleepiness in DM2 to be a discriminative feature between both disorders [49]. Symptoms were rather stable over time in DM1 and DM2 which well corresponds to our neuroimaging data not showing relevant disease-specific progression. These findings underline previous data postulating that fatigue and daytime sleepiness but also apathy in myotonic dystrophies more likely result from central nervous system dysfunction than from respiratory muscle weakness [50]. Nevertheless, a recent randomized controlled trial on fatigued DM1 patients gives reasons to be optimistic, showing that implementation of cognitive behavioural therapy is able to lead to a higher capacity for activity and social participation [51].

Our adult-onset DM1 patients showed only minor areas of gray matter reduction at baseline that were slightly more pronounced at follow-up. However, there were no significant differences in gray matter changes over time between patients and controls in longitudinal analyses. Progressive gray matter loss in DM1 had been assumed earlier according to cross-sectional neuroimaging study results [5, 9, 10, 12, 14].There is discrepancy in the literature about the effect of including congenital DM1 patients on the extent of gray matter changes in neuroimaging analyses [9, 14]. Some data hint to a more pronounced gray matter loss when congenital forms are included, others suggest the opposite.

Frequency and extent of WML were more pronounced in DM1 than in DM2 and in controls. A relatively high prevalence of WML in DM1 had been shown earlier [8, 10, 52]. Intriguingly, WML in our DM patients did not change significantly within the observation period. We found widespread white matter reduction and microstructural white matter alterations at baseline and follow-up that were more pronounced in DM1 than in DM2 compared to controls. The focus on isolated disease-related effects however did not show significant differences in changes of white matter density and microstructure between DM1 and controls over time, and patterns of cerebral involvement remained stable.

In DM2, there was no gray matter reduction at baseline and at follow-up. This conforms to our previous study, indicating reproducible results [10]. However, earlier studies including VBM analyses had revealed gray matter atrophy in DM2 patients [11, 53]. Frequency and extent of WML did not differ from controls and were stable over time. White matter reduction and microstructural alterations in group comparison at follow-up exceeded those observed at baseline, but there were no significant differences in changes over time between DM2 patients and controls. The isolated higher AD increase in controls compared to DM2 over time affected only a small portion of the genu of the corpus callosum and might imply “healthy aging” in controls [54]. Specifically, it may indicate a catch-up effect of healthy subjects in a region known to undergo disease-related alterations in both, DM1 and DM2 patients [10]. White matter changes in general were less pronounced in DM2 than in DM1 and also less severe than in our previous study, potentially on account of smaller sample sizes or drop out of older DM2 patients at follow-up, respectively [10].

The clinical implication of severe white matter changes, more pronounced in DM1 than in DM2 patients, is still not fully understood. In this study, we found slightly more neuropsychological deficits in DM1 than in DM2 patients in our cross-sectional and longitudinal analyses, potentially mirroring the more pronounced white matter alterations in the DM1 compared to the DM2 group. However, correlation analyses performed earlier in our baseline study did not show any correlation of white matter changes with neuropsychological testing results [10]. Nevertheless, other studies did suggest a correlation between diffusivity parameters and cognitive function in DM1 [12, 14]. Equally, as discussed in our baseline study, we found degradation of pathways of the limbic system in DM1 but not in DM2 patients. This could be associated with behavioural abnormalities and specific personality traits observed in DM1 but not in DM2 patients [55]. Serra et al. suggested an association between dysfunctional social cognition in DM1 and abnormal brain connectivity [15]. This could be in line with the hypothesis that a lower degree of white matter alterations in DM2 may account for a lower prevalence and extent of personality changes in this patient group.

## Conclusion

In conclusion, white matter changes were more pronounced in DM1 than DM2 and exceeded gray matter involvement by far. The aetiology of white matter alterations in myotonic dystrophies is not yet fully understood, and systematic post-mortem analyses of brain tissue are not available so far. In particular, the potential role of microvascular pathology is still unclear. In our patient series, vascular risk factors were not more frequent than in our controls. This is remarkable and might underline a more complex and multifactorial origin of white matter abnormalities. However, microvascular changes underlying the observed white matter changes cannot be ruled out completely. Patterns of white matter changes were consistent, highly similar at baseline and follow-up, and also similar to our previous study [10]. This indicates strong, reproducible and robust effects that had been shown previously [8, 9, 11, 13, 14, 52, 53]. Using VBM and DTI difference maps analyses, we could demonstrate that there were no relevant differences in brain morphological changes over time between myotonic dystrophy patients and controls within the observation period. This argues against a pronounced progressing e.g. neurodegenerative process in middle-aged classical adult-onset DM1 and DM2 patients. It might support the hypothesis that white matter changes in adult-onset DM1 and DM2 may be of primarily early-life or even neurodevelopmental origin as previously described in congenital forms of DM1. It needs to be considered that we performed our analyses in middle-aged patients. Thus, brain morphological changes might have occurred post-developmental but earlier in adult-life. On account of an observation period of no more than 6 years, our results would also be consistent with a slowly progressive degenerative disorder or progeroid disease. A previous 3T-H-MRS study in DM1 postulated that brain abnormalities may derive from neurodevelopmental changes rather than from neurodegeneration [56]. However, a recent longitudinal neuropsychological study suggested an accelerated normal aging process [16].

Results should be interpreted with caution due to sample sizes and a restricted observation period. Furthermore, it has to be questioned if the applied MRI methods are sensitive enough and provide sufficient statistical power to be able to detect microstructural brain alterations in comparably small sample sizes. Nevertheless, neuroimaging studies on multiple sclerosis patients had been able to detect differences in brain atrophy over a timespan of only four years by examination of very small patient numbers [57].

Larger patient groups and longer observation periods are particularly difficult to realize in rare diseases like myotonic dystrophies associated with reduced lifespans. Better age-matched controls would also have been desirable for the comparison with DM1 patients. Unfortunately, recruiting the same healthy controls in a longitudinal study is particularly challenging, and we addressed age differences between groups by including age as covariate in our statistical design.

It is of high importance to clarify the nature and natural history of brain involvement against the background of currently upcoming specific treatment studies in DM1. A most clear definition of cerebral changes and its surrogate markers is of urgent need not only for the design of therapy compounds but also for the identification of uniform study protocols, biomarkers, and appropriate clinical trial outcome parameters [17, 18]]. Our imaging findings contribute to the natural history data on myotonic dystrophies and highlight the rather slow progression of morphological brain alterations in middle-aged patients. This should be taken into account in future treatment trials.

## Supporting information

S1 Table Mean FA values ± SD of altered cerebral regions in patients and controls. Given values are mean FA values ± SD of those voxels within fiber tracts that showed significantly reduced FA in patients in comparison to controls at baseline (T1) and within identical regions at follow-up (T2). SLF = superior longitudinal fascicle, ILF = inferior longitudinal fascicle, IFOF = inferior fronto-occipital fascicle, UF = uncinate fascicle, CGB = cingulum bundle
(DOCX)

S2 Table Demographic, clinical and neuropsychological data. Raw values of all demographic, clinical, and neuropsychological data of each participant obtained in this study are given. To reduce the risk of personal identification, we omitted any information regarding sex and provide instead of the precise age an individual age range (3 = 31–40 years; 4 = 41–50 years; 5 = 51–60 years; 6 = 61–70 years, 7 = 71–80 years).
(XLSX)
