# Supplementary material for: Tracking the brain in myotonic dystrophies: A 5-year longitudinal follow-up study
Source: PLoS One. 2019 Mar 7;14(3):e0213381. doi: 10.1371/journal.pone.0213381 (PMC6405094; doi:10.1371/journal.pone.0213381)
Supplement: S1 Table — Given values are mean FA values ± SD of those voxels within fiber tracts that showed significantly reduced FA in patients in comparison to controls at baseline (T1) and within identical regions at follow-up (T2). SLF = superior longitudinal fascicle, ILF = inferior longitudinal fascicle, IFOF = inferior fronto-occipital fascicle, UF = uncinate fascicle, CGB = cingulum bundle (DOCX) [file pone.0213381.s001.docx]

**S1 Table. Mean FA values ± SD** **of altered cerebral regions in patients and controls.**

| **DM1** |  |  |  |  |  | |
| --- | --- | --- | --- | --- | --- | --- |
| **fiber tract** | **side** | **mean FA values**  **DM 1** | |  | **mean FA values**  **controls** | |
|  |  | **T1** | **T2** |  | **T1** | **T2** |
|  |  |  |  |  |  |  |
| SLF | right | 0.410 ± 0.028 | 0.290 ± 0.023 |  | 0.503 ± 0.025 | 0.355 ± 0.020 |
|  | left | 0.436 ± 0.031 | 0.308 ± 0.025 |  | 0.538 ± 0.029 | 0.374 ± 0.020 |
|  |  |  |  |  |  |  |
| ILF | right | 0.425 ± 0.032 | 0.305 ± 0.028 |  | 0.527 ± 0.025 | 0.372 ± 0.017 |
|  | left | 0.434 ± 0.034 | 0.314 ± 0.027 |  | 0.539 ± 0.027 | 0.382 ± 0.020 |
|  |  |  |  |  |  |  |
| IFOF | right | 0.422 ± 0.031 | 0.313 ± 0.025 |  | 0.523 ± 0.026 | 0.384 ± 0.019 |
|  | left | 0.412 ± 0.032 | 0.303 ± 0.026 |  | 0.512 ± 0.026 | 0.372 ± 0.018 |
|  |  |  |  |  |  |  |
| UF | right | 0.387 ± 0.028 | 0.262 ± 0.021 |  | 0.491 ± 0.025 | 0.327 ± 0.017 |
|  | left | 0.365 ± 0.031 | 0.261 ± 0.024 |  | 0.459 ± 0.024 | 0.327 ± 0.015 |
|  |  |  |  |  |  |  |
| forceps min. |  | 0.423 ± 0.035 | 0.292 ± 0.025 |  | 0.527 ± 0.030 | 0.363 ± 0.019 |
|  |  |  |  |  |  |  |
| forceps maj. |  | 0.495 ± 0.025 | 0.426 ± 0.029 |  | 0.594 ± 0.022 | 0.511 ± 0.026 |
|  |  |  |  |  |  |  |
| cingulum | right | 0.490 ± 0.034 | 0.385 ± 0.030 |  | 0.589 ± 0.027 | 0.452 ± 0.024 |
|  | left | 0.484 ± 0.032 | 0.400 ± 0.028 |  | 0.584 ± 0.028 | 0.480 ± 0.024 |
|  |  |  |  |  |  |  |
| corpus callosum |  | 0.635 ± 0.033 | 0.577 ± 0.034 |  | 0.737 ± 0.019 | 0.669 ± 0.019 |
|  |  |  |  |  |  |  |
| external capsule | right | 0.441 ± 0.025 | 0.380 ± 0.033 |  | 0.532 ± 0.022 | 0.458 ± 0.025 |
|  | left | 0.413 ± 0.034 | 0.363 ± 0.035 |  | 0.510 ± 0.025 | 0.440 ± 0.030 |
|  |  |  |  |  |  |  |
| internal capsule | right | 0.540 ± 0.032 | 0.468 ± 0.030 |  | 0.629 ± 0.040 | 0.510 ± 0.035 |
|  | left | 0.570 ± 0.024 | 0.536 ± 0.030 |  | 0.657 ± 0.030 | 0.600 ± 0.039 |
|  |  |  |  |  |  |  |
| int. capsule (retr.lent.part) | right | 0.501 ± 0.042 | 0.390 ± 0.031 |  | 0.596 ± 0.033 | 0.458 ± 0.028 |
|  | left | 0.516 ± 0.035 | 0.438 ± 0.038 |  | 0.607 ± 0.027 | 0.502 ± 0.026 |
|  |  |  |  |  |  |  |
| corticospinal tract | right | 0.490 ± 0.036 | 0.363 ± 0.027 |  | 0.570 ± 0.035 | 0.422 ± 0.031 |
|  | left | 0.474 ± 0.030 | 0.297 ± 0.022 |  | 0.558 ± 0.028 | 0.347 ± 0.021 |
|  |  |  |  |  |  |  |
| **DM2** |  |  |  |  |  |  |
| **fiber tract** | **side** | **mean FA values**  **DM2** | |  | **mean FA values**  **controls** | |
|  |  | **T1** | **T2** |  | **T1** | **T2** |
|  |  |  |  |  |  |  |
| corpus callosum | left | 0.454 ± 0.039 | 0.356 ± 0.030 |  | 0.533 ± 0.037 | 0.407 ± 0.037 |
|  |  |  |  |  |  |  |
| forceps min. | left | 0.373 ± 0.031 | 0.269 ± 0.029 |  | 0.440 ± 0.035 | 0.304 ± 0.035 |
